# HIF-1α activation results in actin cytoskeleton reorganization and modulation of Rac-1 signaling in endothelial cells

**DOI:** 10.1186/1478-811X-11-80

**Published:** 2013-10-21

**Authors:** Alexander Weidemann, Johannes Breyer, Margot Rehm, Kai-Uwe Eckardt, Christoph Daniel, Iwona Cicha, Klaudia Giehl, Margarete Goppelt-Struebe

**Affiliations:** 1 Department of Nephrology and Hypertension, Universitätsklinikum Erlangen, Universität Erlangen-Nürnberg, Loschgestrasse 8, 91054 Erlangen, Germany; 2 Department of Nephropathology, Universitätsklinikum Erlangen, Universität Erlangen-Nürnberg, Krankenhausstrasse 8-10, 91054 Erlangen, Germany; 3 Department of Cardiology and Angiology, Universitätsklinikum Erlangen, Universität Erlangen-Nürnberg, Schwabachanlage 10, 91054 Erlangen, Germany; 4 Molecular Oncology of Solid Tumors, Internal Medicine IV/V, Justus-Liebig-University Giessen, Schubertstr. 81 – BFS, 35392 Giessen, Germany; 5 Present address: Department of Urology, University of Regensburg, Regensburg, Germany

**Keywords:** Hypoxia-inducible transcription factor, endothelial cell, cytoskeleton, migration, Rho kinase, Rac-1, p21-activated kinase, prolyl hydroxylase inhibitor, von Hippel-Lindau protein, oxygen

## Abstract

**Background:**

Hypoxia is a major driving force in vascularization and vascular remodeling. Pharmacological inhibition of prolyl hydroxylases (PHDs) leads to an oxygen-independent and long-lasting activation of hypoxia-inducible factors (HIFs). Whereas effects of HIF-stabilization on transcriptional responses have been thoroughly investigated in endothelial cells, the molecular details of cytoskeletal changes elicited by PHD-inhibition remain largely unknown. To investigate this important aspect of PHD-inhibition, we used a spheroid-on-matrix cell culture model.

**Results:**

Microvascular endothelial cells (glEND.2) were organized into spheroids. Migration of cells from the spheroids was quantified and analyzed by immunocytochemistry. The PHD inhibitor dimethyloxalyl glycine (DMOG) induced F-actin stress fiber formation in migrating cells, but only weakly affected microvascular endothelial cells firmly attached in a monolayer. Compared to control spheroids, the residual spheroids were larger upon PHD inhibition and contained more cells with tight VE-cadherin positive cell-cell contacts. Morphological alterations were dependent on stabilization of HIF-1α and not HIF-2α as shown in cells with stable knockdown of HIF-α isoforms. DMOG-treated endothelial cells exhibited a reduction of immunoreactive Rac-1 at the migrating front, concomitant with a diminished Rac-1 activity, whereas total Rac-1 protein remained unchanged. Two chemically distinct Rac-1 inhibitors mimicked the effects of DMOG in terms of F-actin fiber formation and orientation, as well as stabilization of residual spheroids. Furthermore, phosphorylation of p21-activated kinase PAK downstream of Rac-1 was reduced by DMOG in a HIF-1α-dependent manner. Stabilization of cell-cell contacts associated with decreased Rac-1 activity was also confirmed in human umbilical vein endothelial cells.

**Conclusions:**

Our data demonstrates that PHD inhibition induces HIF-1α-dependent cytoskeletal remodeling in endothelial cells, which is mediated essentially by a reduction in Rac-1 signaling.

## Introduction

Hypoxia is a major driving force in vascularization and vascular remodeling [1]. Many of the genes involved in the regulation of vascular homeostasis are direct or indirect targets of hypoxia-induced transcription factors (HIFs) known as crucial mediators of the cellular response to hypoxia [2,3]. HIF is a heterodimeric DNA-binding complex composed of the constitutive non-oxygen-responsive subunit HIF-1β, and one of either hypoxia-inducible α-subunits, HIF-1α or HIF-2α [4]. HIF-α subunits are rapidly degraded in normoxia but highly stabilized by hypoxia. In normoxia, two prolyl residues of the HIF-α subunits are hydroxylated by prolyl hydroxylase domain enzymes (PHDs), which belong to the group of iron- and 2-oxoglutarate-dependent oxygenases [5]. This modification is the prerequisite for binding of the von Hippel-Lindau tumor suppressor protein (pVHL), which targets HIF-α for proteasomal degradation. Genetic studies in mice have shown that deletion of Vhl in various tissues leads to a pathologically altered vascular phenotype through the constitutive activation of the hypoxia response pathway [6]. In contrast, partial inhibition of HIF degradation in heterozygous PHD2 deficient mice led to normalization of endothelial lining and vessel maturation in tumor vessels [7].

Angiogenesis as a response to activation of HIF transcription factors has been investigated in many studies related to tumor vascularization (for review see [1,8,9]) but also in context of atherosclerosis [10], or wound healing [11]. Accordingly, inhibition of PHDs by small molecules as a way to target remodeling of the vasculature has recently attracted increasing attention [12-14]. Various preclinical studies showed promising effects of PHD inhibitors: in a mouse model of skeletal trauma, PHD inhibition increased vascularity and subsequently callus formation [15]. After myocardial infarction, inhibition of PHDs improved microvascular density in the periinfarct region [16], which was also observed in the ischemic brain of mice treated with dimethyloxalyl glycine (DMOG) following experimental stroke [17]. PHD inhibitors increased endothelial cell migration from spheroids in three dimensional collagen gels in vitro [18,19] or in angiogenesis assays such as the sponge model [15,20]. However, apart from HIF-dependent transcriptional regulation of angiogenic factors, molecular effects of PHD inhibition on the vasculature have not been studied extensively.

Vascular remodeling implies reorganization of the actin cytoskeleton of endothelial cells [21]. Small GTPases of the Rho family are major regulators of the actin cytoskeleton, and vascular permeability has been shown to be controlled by Rho family proteins, particularly RhoA and Rac1 [22,23]. Activation of Rac1 was linked to HIF-1α activation and stabilization in endothelial cells [24,25] and vascular smooth muscle cells [26]. By contrast, little is known about HIF-induced alterations in GTPase-mediated remodeling of actin filaments in endothelial cells. Transient alterations in F-actin fibers were observed within 1 h of exposure to hypoxia in pulmonary endothelial cells, which reverted to normal after 2 h [27] or even decreased compared to cells cultured in normoxia [28]. Long term activation of HIF as achieved by pharmacological inhibition of PHDs has not yet been studied in endothelial cells in terms of actin remodeling.

In this study, we addressed the question of how inhibition of PHDs by DMOG affects actin cytoskeletal organization in microvascular endothelial cells. In an earlier study, we had observed that renal microvascular endothelial cells (glEND.2) seeded on glass plates or matrix coated surfaces hardly migrated in conventional barrier or scratch assays [19]. Therefore, we modified the model system and organized the cells into spheroids, which were then plated on matrix-coated plates, where the cells migrated off the spheroids within 24 h. This model system addressed two aspects of endothelial cell interactions: three dimensional homotypic cell-cell interactions within the spheroids, and cell-matrix interactions upon migration.

By using this system, we show that inhibition of PHDs by DMOG increased cell-cell attachment within the spheroids and strengthened F-actin stress fibers in migrating endothelial cells outside the spheroids. Using stable HIF-1α- or HIF-2α-deficient glomerular endothelial cells, we demonstrate that cytoskeletal alterations by PHD inhibition are HIF-1α dependent. DMOG modulated the subcellular localization of Rac-1 and activation of its downstream target p21-activated kinase (PAK). Taken together, our data provides the first evidence of a link between pharmacological inhibition of PHDs and cytoskeletal rearrangement and migration of endothelial cells.

## Results

### DMOG modulates endothelial migration and cell-cell contacts within spheroids

**Figure 1:**
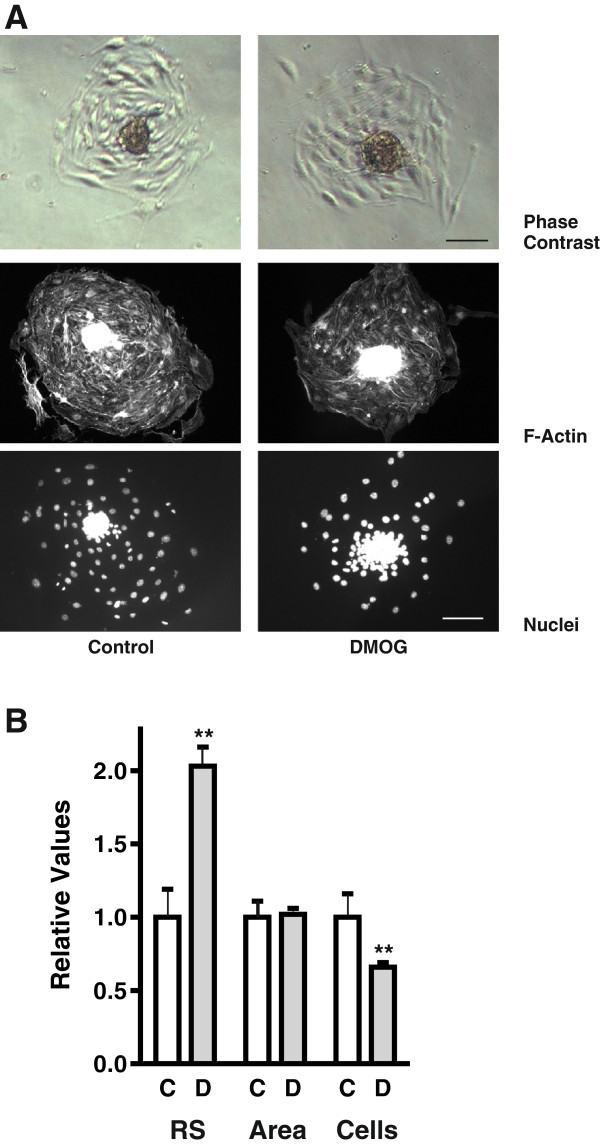
**DMOG alters cell-cell and cell-matrix interactions.** Spheroids of microvascular endothelial glEND.2 cells were generated as described in Materials and Methods. Upon plating on collagen IV-coated plates cells were treated with 1 mM DMOG for 24 h. **(A)** Radial migration off the spheroids after 24 h was significantly impaired by DMOG-treatment and more cells remained in the residual spheroids. F-actin fibers were visualized with PromoFluor phalloidin, nuclei were stained with Hoechst. Scale bar: 100 μm. **(B)** For quantification at least 6 spheroids were analyzed and the data of 7 independent experiments were summarized as means ± SEM, **p < 0.01, one sample *t*-test. C: Control cells; D: DMOG-treated cells; RS: area covered by residual spheroids; Area: area covered by migrated cells; Cells: number of cells migrated off the spheroids.

Murine glomerular microvascular endothelial cells (cell line glEND.2) were organized into spheroids overnight and were then plated on glass plates coated with collagen IV in the presence or absence of the PHD inhibitor DMOG, which leads to the stabilization of HIF-α transcription factors. Approximately 5 h after adherence, cells started to migrate radially from the spheroids. Most of the cells migrated as a cohort with only few cells loosing contact (Figure 1A). Movement of the cells was monitored using live cell imaging. DMOG-treated cells moved in a directional fashion and extended large protrusions, whereas protrusions were much smaller in control cells, which closely interacted with each other and even crawled below each other thus appearing more motile (Additional file 1).

Staining of F-actin fibers with PromoFluor phalloidin allowed quantification of the area covered by cells still organized in residual spheroids as well as the area covered by migrating cells. The number of migrated cells was determined after nuclear staining with DAPI. After 24 h, the size of the residual spheroids (RS) of DMOG-treated cells was significantly larger than that of control cells (Figure 1B). Correspondingly, the number of cells which had migrated from the spheroids was markedly reduced. However, the area covered by the cohesively migrating cells was comparable under both conditions, indicative of a larger area covered by individual cells in the DMOG group. A comparable effect was observed when endothelial cells were plated in a cell culture dish as subconfluent cells in the presence of DMOG. After 24 h, the area covered by individual DMOG-treated cells was about 30% larger than that of control cells (Additional file 2: Figure S2A/B).

### DMOG leads to long-term alterations of F-actin structures

**Figure 2:**
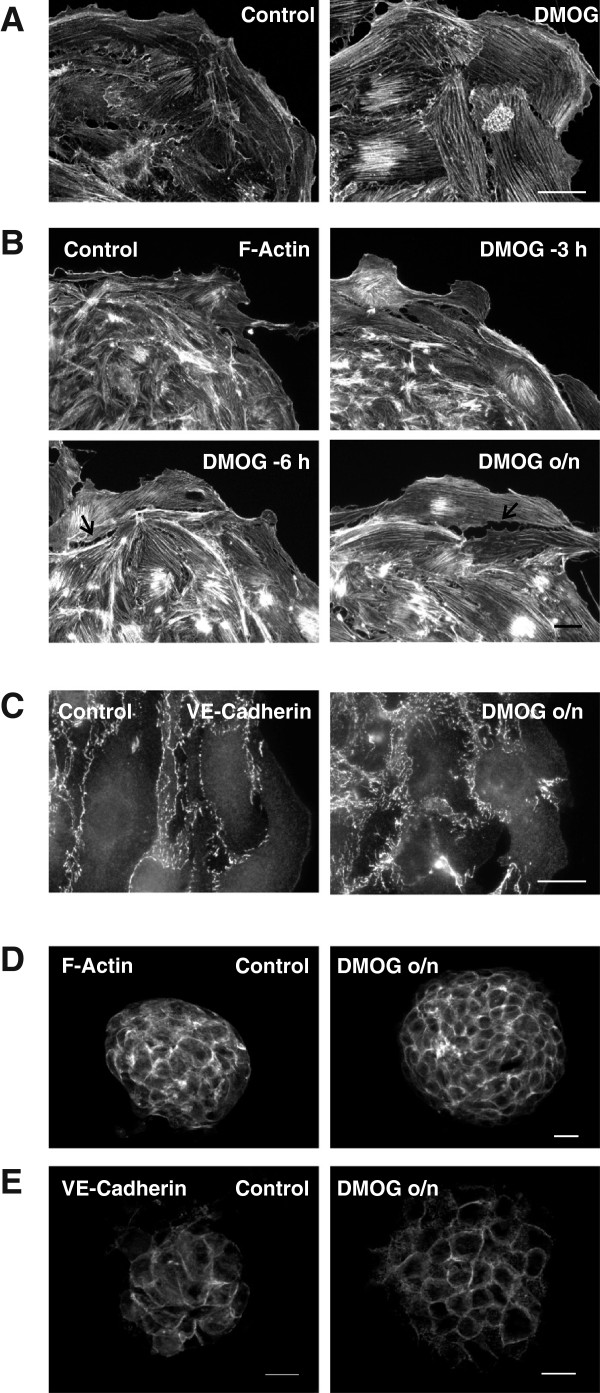
**DMOG strengthens F-actin fibers and supports cell structures within spheroids. (A)** DMOG strongly affected orientation of F-actin fibers in migrating cells treated with 1 mM DMOG for 24 h. Compared to control cells, a regular pattern of strong parallel F-actin fibers was observed (PromoFluor phalloidin staining, confocal microscopy). Scale bar: 20 μm. **(B)** F-actin fibers were still highly irregular when cells were treated with 1 mM DMOG for the last 3 h only (DMOG -3 h), whereas treatment with DMOG for the last 6 h induced alignment of F-actin fibers (DMOG -6 h). Arrows indicate gaps between cells. Scale bar: 20 μm. **(C)** Cell-cell contacts were visualized by staining of VE-cadherin after spheroids were treated with 1 mM DMOG for 24 h. Scale bar: 20 μm. **(D/E)** F-actin structures **(D)** and VE-cadherin **(E)** within the spheroids were visualized by apotome technique. Cortical F-actin and VE-cadherin formed distinct patterns in DMOG-treated cells. Scale bar: 20 μm.

Treatment with DMOG over night markedly altered the network of the F-actin fibers of migrating cells (Figure 2A). Control cells showed a rather irregular pattern of F-actin fibers, whereas DMOG-treated cells were characterized by thick F-actin fibers oriented in parallel. When cells were treated with DMOG for the last 6 h or 3 h of the migration period of 24 h, it became evident that 6 h were needed to induce aligned F-actin fibers (Figure 2B). After treatment for 3 h DMOG-treated cells still resembled control cells, even though earlier experiments had shown that HIF-1α was stabilized by DMOG within 1 h [29] and rearrangement of F-actin fibers may occur within 1 h upon stimulation with lysophosphatidic acid (data not shown). These findings led to the hypothesis that rearrangement of F-actin fibers was not due to DMOG per se, but required HIF-1α-dependent regulation of protein synthesis. Treatment with DMOG not only increased the area covered by individual cells but also led to gaps between individual cells, possibly reflecting the tension within the cells due to the strong cell spanning F-actin fibers (Arrow heads in Figure 2B, lower panels).

Treatment with DMOG markedly altered VE-cadherin mediated cell-cell adhesions (Figure 2C). While linear distribution of VE-cadherin along the cell borders prevailed in control cells, orthogonal orientation of VE-cadherin was characteristic of DMOG-treated cells.

Within the spheroids the endothelial cells showed peripheral F-actin fibers typical for cells cultured in three-dimensional conditions (Figure 2D). DMOG-treated spheroids appeared more organized, as also shown by the well-ordered VE-cadherin structures (Figure 2E). Nuclei within the spheroids remained structurally intact and did not show any signs of necrosis or apoptosis due to hypoxia or shortage of nutrients (data not shown). DMOG also modified the structural organization of spheroids when added during the time of spheroid generation as hanging drops. Spheroids obtained in the presence of DMOG appeared more compact than control spheroids (Additional file 3: Figure S3A/B).

Next, we compared the impact of DMOG on endothelial cell morphology in cells migrating from spheroids with the effects of DMOG on cells cultured as monolayers. DMOG treatment of subconfluent cells, which had not established firm interactions with other cells and the matrix, induced formation of thick F-actin fibers (Additional file 2: Figure S2B). Changes in cytoskeletal architecture were less obvious when a confluent monolayer of cells was treated with DMOG (Additional file 2: Figure S2C). These observations indicated that in terms of morphological alterations DMOG affected motile cells more strongly than firmly attached cells.

### Morphological alterations are HIF-1α dependent

**Figure 3:**
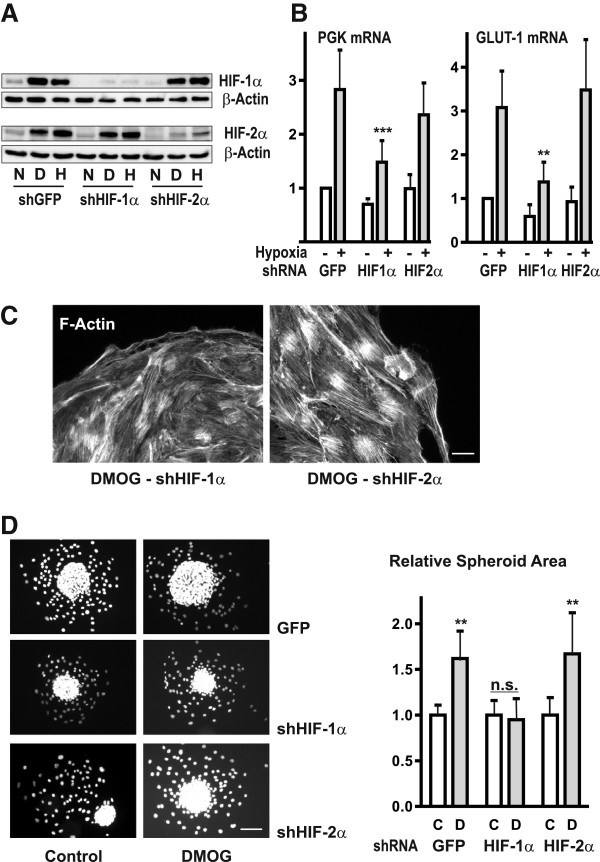
**Morphological alterations induced by DMOG are HIF-1α dependent. (A)** glEND.2 cells were stably transfected with shGFP, shHIF-1α or shHIF-2α. The clones were cultured under normoxic (N) or hypoxic (H, 1% O_2_) conditions, or treated with DMOG (D, 1 mM) for 6 h. 30 μg protein of cellular protein were loaded for the detection of HIF-1α, and nuclear extracts (20 μg protein) were used for the detection of HIF-2α. **(B)** Stably transfected cells, shGFP (GFP), shHIF-1α (HIF1α) or shHIF-2α (HIF2α), were exposed to hypoxia for 20 h. Expression of HIF-target genes phosphoglycerate kinase (PGK) and glucose transporter GLUT-1 mRNA was upregulated in shGFP and shHIF-2α clones but not in shHIF-1α clones. RNA was detected by real time RT-PCR. Expression of PGK or GLUT-1 in GFP cells was set to 1 in each experiment. Data are means ± SD of 4 to 9 experiments. *** p < 0.001, ** p < 0.01 compared to shGFP cells, ANOVA with Tukey’s multiple comparison test. **(C)** Spheroids obtained form glEND2 cells stably transfected with GFP, shHIF-1α or shHIF-2α were plated on fibronectin and treated with 1 mM DMOG for 24 h. Knockdown of HIF-1α prevented formation of strong F-actin fibers as visualized with PromoFluor phalloidin in shHIF-1α clones. Representative images of shHIF-1α or shHIF-2α cells are depicted. Scale bar: 20 μm. **(D)** To quantify the areas covered by residual spheroids, cells were stained with Hoechst. Scale bar: 50 μm Areas were quantified in 2 independent experiments with at least 8 spheroids in each group. Data are summarized as means ± SD; mean value of spheroids of control cells was set to 1 in each experiment; error bars of control values represent variability within one experiment. ** p < 0.01; ANOVA with Dunnett’s multiple comparison test.

The HIF-α isoforms HIF-1α and HIF-2α have been shown to activate different target genes depending on the cellular background [30]. To address the question which HIF-α isoform was responsible for the effects of DMOG, we generated glEND.2 cells with a stable knockdown of HIF-1α and HIF-2α respectively (shHIF-1α and shHIF-2α). Successful generation of those cell lines was demonstrated by knockdown of HIF-α proteins, which were no longer stabilized when the cells were cultured under hypoxic conditions (1% oxygen) or treated with DMOG (Figure 3A). Exposure to hypoxia induced mRNA expression of HIF target genes such as phosphoglycerate kinase PGK and the glucose transporter GLUT1 (Figure 3B). Both were regulated by HIF-1α as shown by the reduced expression in shHIF-1α cells.

We then determined which HIF-α isoform was responsible for the DMOG-dependent morphological alterations of cells migrating from spheroids. F-actin staining of non-stimulated shRNA knockdown cell lines did not differ significantly from control cells (data not shown). Treatment with DMOG strengthened F-actin structures in shHIF-2α clones, but not in shHIF-1α clones, indicating a role for HIF-1α in DMOG-mediated structural alterations (Figure 3C). Concomitantly, DMOG increased the residual spheroid area in GFP-transfected cells and in HIF-2α knockdown cells, but not in cells with stable knockdown of HIF-1α (Figure 3D). These results clearly demonstrated that stabilization of residual spheroids and F-actin alterations by DMOG were HIF-1α dependent.

### Activity of Rho-kinases is necessary, but not sufficient for DMOG-mediated stabilization of glEND.2 cells

**Figure 4:**
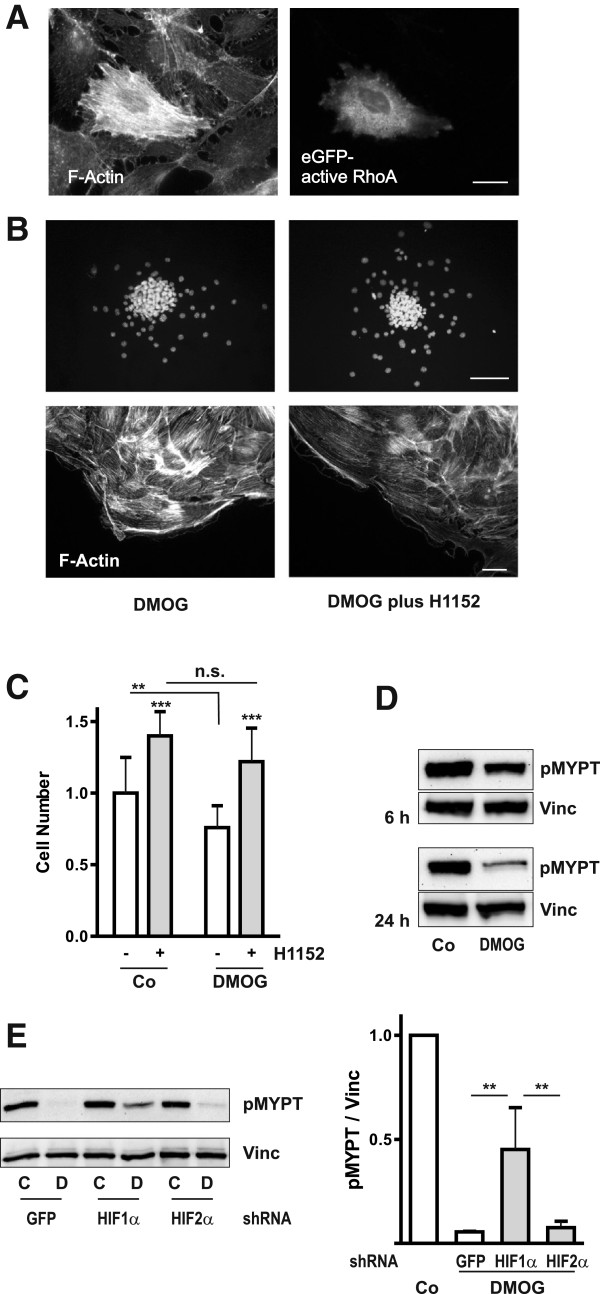
**RhoA - Rho kinase signaling is active in DMOG-treated cells. (A)** glEND.2 microvascular cells cultured in cell culture dishes were transfected with constitutively active eGFP-V14-RhoA. After 24 h, cells were stained for F-actin. Transfected cells are characterized by high density of F-actin fibers. Scale bar: 20 μm. **(B)** Spheroids were treated with DMOG (1 mM) or DMOG plus H1152 (0.75 μM) for 24 h. The Rho kinase inhibitor reduced the size of the remaining spheroids and prevented stress fiber formation. Nuclei were stained with Hoechst, and F-actin fibers with PromoFluor phalloidin. Scale bar upper panel: 100 μm; lower panel: 20 μm. **(C)** The number of cells migrated off spheroids was determined in two independent experiments with at least 15 spheroids for each condition. Mean cell numbers (means ± SD) of control cells was set to 1, error bars of control values represent variability within one experiment. ** p < 0.01 as indicated, *** p < 0.001 compared to respective controls, n.s: not significant; ANOVA with Dunnett’s multiple comparison test. **(D)** Cells cultured in cell culture dishes were incubated with DMOG for 6 and 24 h. Phosphorylated MYPT was detected in cell culture homogenates by Western blotting. The blot is representative of three experiments. **(E)** glEND2 cells stably transfected with GFP, shHIF-1α or shHIF-2α were cultured in cell culture dishes with (D) or without (C) DMOG for 24 h. Phosphorylated MYPT was detected by Western blotting. For quantification, the ratio pMYPT/Vinc of non-treated cells was set to 1 at each experimental condition (Co). ** p < 0.01, n = 3, means ± SD, ANOVA with Tukey’s multiple comparison test.

Formation of intracellular stress fibers is largely regulated by the activity of the small GTPase RhoA and Rho kinases. To analyze the effect of a long lasting activation of RhoA signaling, constitutively active RhoA was overexpressed in glEND.2 cells. Transfected cells formed a dense network of fine F-actin fibers (Figure 4A). Due to the poor transfectability of the microvascular endothelial cells only few individual cells overexpressed constitutively active RhoA and therefore, overexpression of RhoA did not affect cell migration or spheroid size. Cells overexpressing dominant negative RhoA (dnRhoA) rounded, lost cell-cell contacts with neighboring cells and detached, thus preventing further analysis of F-actin structures (data not shown). Therefore, we pharmacologically inhibited Rho effectors, namely Rho-kinases by H1152, which completely prevented the formation of F-actin stress fibers and also reduced the size of the residual spheroids in DMOG-treated cells (Figure 4B). Cells within the spheroids appeared less tightly packed, suggesting a decrease in cell-cell adhesions. We have shown earlier that inhibition of Rho kinases increased directional motility of microvascular endothelial cells, reflected among others in increased numbers of migrated endothelial cells from spheroids [19]. This was also observed in the presence of DMOG: coincubation with H1152 significantly increased the number of migrating cells (Figure 4C). This data provides evidence that Rho kinase activity was necessary to maintain the DMOG-induced morphological alterations, and also supported the cell-cell adherence within the spheroid.

However, incubation of the cells with DMOG for 6 h led to a moderate decrease in Rho kinase activity as determined by phosphorylation of the substrate MYPT (myosin phosphatase target protein) (Figure 4D). A further decrease of Rho kinase activity was observed after 24 h of treatment. A comparable decrease in MYPT phosphorylation was detected in shGFP and shHIF-2α transfected cells, whereas inhibition was significantly less pronounced in shHIF-1α clones (Figure 4E). This indicated that inhibition of MYPT phosphorylation by DMOG was regulated by HIF-1α.

Taken together, these data revealed two aspects of DMOG-mediated structural reorganization of endothelial cells. On the one side, intact Rho kinase signaling is critical for the DMOG-mediated cytoskeletal alterations. On the other hand, in the context of DMOG-induced interference with enzymes regulating actin structures Rho kinase activity itself is dependent on HIF-1α.

### Rac-1 signaling is reduced by DMOG in a HIF-1α-dependent manner

**Figure 5:**
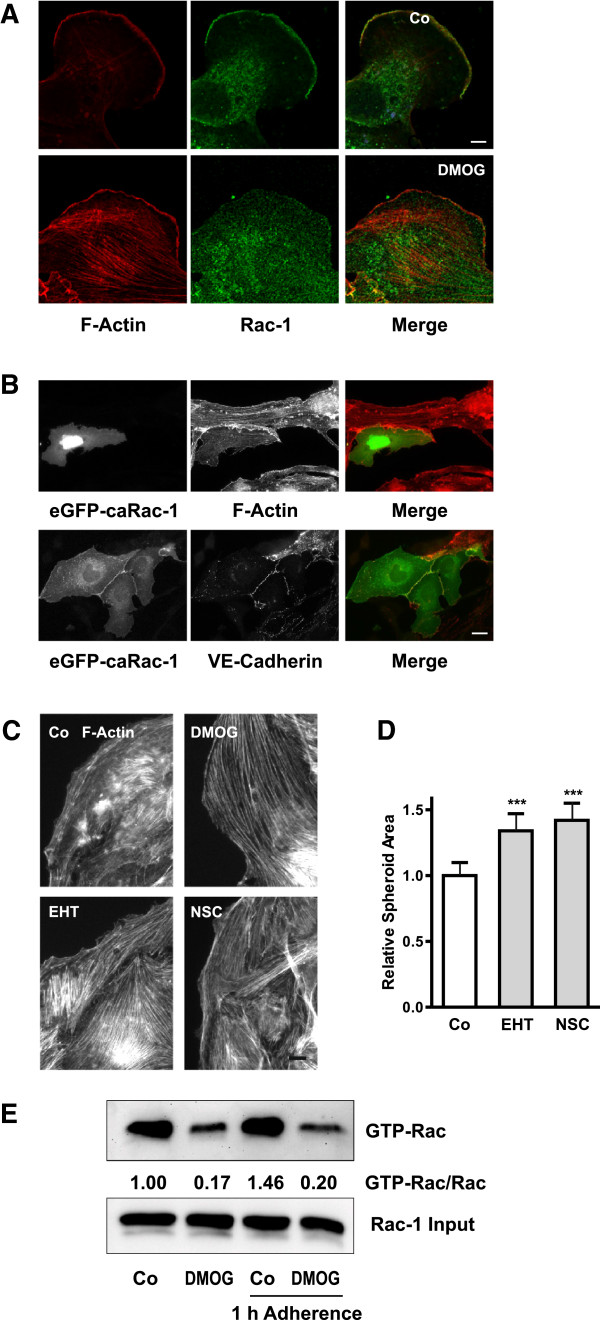
**Rac-1 activity is reduced in DMOG-treated glEND.2 cells. (A)** Spheroids were treated with 1 mM DMOG overnight. F-actin (red) and VE-cadherin (green) were visualized by PromoFluor phalloidin staining and indirect immunofluorescence, respectively, using confocal microscopy. Colocalization at the cell periphery of migrating cells was observed in control cells but not in DMOG-treated cells. Scale bar: 5 μm. **(B)** glEND.2 cells cultured in cell culture dishes were transfected with constitutively active eGFP-V12-Rac-1 coupled to GFP. Cells were analyzed after 24 h. Overexpression of Rac-1 reduced F-actin stress fibers (upper panels). Rac-1 was concentrated at the cell borders as shown by colocalization with VE-Cadherin (lower panels). Scale bar: 20 μm. **(C)** Spheroids were treated with DMOG (1 mM), EHT1864 (EHT, 5 μM) or NSC23677 (NSC, 100 μM) for 24 h leading to strengthening of F-actin fibers. F-actin was visualized by PromoFluor phalloidin staining. Scale bar: 10 μm. **(D)** Residual spheroid area was determined after 24 h in 3 independent experiments with 9–10 spheroids for each condition; mean value of spheroid areas in control cells was set to 1 in each experiment; means ± SD *** p < 0.001 compared to control cells, Dunnett’s post hoc test. **(E)** glEND.2 cells treated with DMOG (1 mM) were cultured in cell culture dishes over night and then were reseeded for 1 h. GTP-Rac was precipitated from 500 μg cellular protein. Total Rac was detected in 25 μg cellular protein. The ratios Rac-GTP/Rac (given between the blots) show a strong decrease in samples treated with DMOG. The blot is representative of two independent experiments with a reduction of Rac-GTP > 80%.

Actin structures are largely dependent on the equilibrium between activities of Rho and Rac GTPases. To assess the role of Rac-1 activity in cells migrating out of spheroids, Rac-1 localization was detected by immunocytochemistry. Lamellipodia of migrating endothelial cells frequently showed Rac-1 colocalizing with peripheral F-actin (Figure 5A, upper panel). In DMOG-treated cells, Rac-1 was barely detectable at the periphery (Figure 5A, lower panel). Therefore, we investigated whether overexpression of dominant negative Rac-1 (dnRac-1) could mimic the changes in F-actin structures observed upon DMOG treatment. However, overexpression of dnRac-1 resulted in apoptotic cell death, as obvious by altered nuclear morphology (data not shown). In contrast, overexpression of constitutively active Rac-1 resulted in cells which were almost void of intracellular F-actin fibers (Figure 5B, upper panel). Moreover, constitutively active Rac-1 co-localized with VE-cadherin at the cell-cell junctions (Figure 5B, lower panel).

To circumvent the problem of poor transfectability and cell death by dnRac-1, cells were treated with two chemically distinct Rac inhibitors, EHT1864 and NSC23677, which also differ in their molecular targeting [31,32]. Incubation of spheroids with EHT1864 or NSC23677 induced the formation of cell spanning F-actin fibers which resembled those obtained in the presence of DMOG (Figure 5C). Compared to DMOG, however, the thickness of the fibers was reduced, in line with the notion that DMOG-mediated stabilization of HIF-1α goes beyond modulation of Rac-1. Pharmacological inhibition of Rac-1 also increased the size of the residual spheroids indicative of stabilization of cell-cell adhesions (Figure 5D). In line with Rac-1 being located downstream of HIF, residual spheroids were increased in both, shHIF-1α and shHIF-2α clones upon incubation with NSC23677 (Additional file 4: Figure S4). Rac-1, however, was not a direct transcriptional target of HIF-α. Western blot analysis of cells, which were freshly seeded and reacted to DMOG with morphological alterations, did not alter Rac-1 expression as determined by Western blotting (data not shown), indicative of regulation of Rac-1 activity, but not Rac-1 protein. This was confirmed by direct assessment of the amount of GTP-bound active Rac-1, which was downregulated after overnight treatment with DMOG and remained inactive upon seeding of the cells (Figure 5E).

### PAK is inhibited by DMOG in a HIF-1α-dependent manner

**Figure 6:**
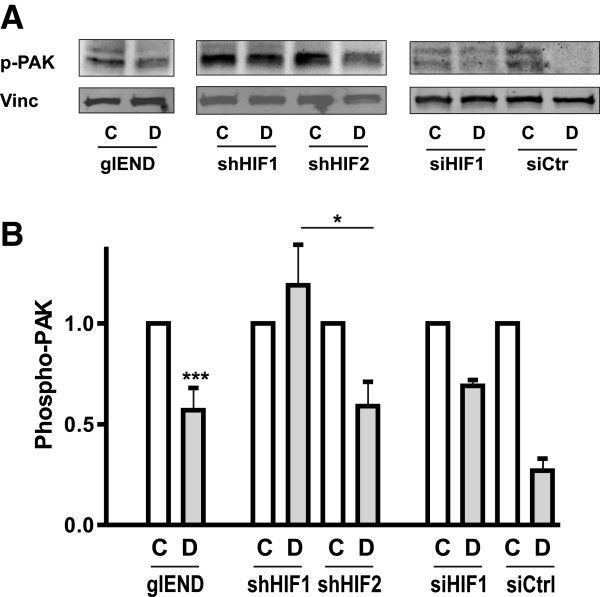
**PAK phosphorylation is reduced by DMOG in a HIF-1α dependent fashion. (A)** glEND.2 cells were incubated with 1 mM DMOG over night and were then replated for 45 to 60 min. Endogenous phospho-PAK was detected by Western blotting. Blots show representative experiments. **(B)** The graph depicts the ratio phospho-PAK to vinculin detected in different experiments. Downregulation of HIF-1α by shRNA or siRNA prevented DMOG-mediated reduction of PAK phosphorylation as observed in control cells or shHIF-2α clones. Value of control cells was set to 1 in each experiment. glEND.2: n = 5, *** p < 0.001, two sided one sample *t*-test; shHIF: n = 3, p < 0.05, Student’s *t*-test; siHIF: means ± half range of two experiments.

To further delineate Rac-1 signaling, we analyzed the expression and activation of endogenous activated p21-activated kinase (PAK). PAK is directly activated by Rac-1 and mediates many of its morphological effects in endothelial cells [33]. To quantify phosphorylated PAK, Rac-1/PAK signaling was activated by cell adhesion. Cells were incubated with DMOG over night and then replated for 1 h. Phosphorylated endogenous PAK was detectable in control cells and was downregulated in DMOG-treated cells (Figure 6) Comparison of shHIF-1α and shHIF-2α clones showed reduced PAK activity in DMOG-treated cells only in shHIF-2α clones, which indicated that HIF-1α was necessary for PAK regulation. To confirm the role of HIF-1α in PAK inhibition and rule out effects of chronic HIF-1α knockdown, HIF-1α was transiently silenced by specific siRNA as described previously [29]. As expected, siHIF-1α inhibited DMOG-mediated reduction of PAK phosphorylation (Figure 6, right panel). Taken together, these results demonstrate that functional HIF-1α is necessary for the inhibition of phosphorylation of PAK by DMOG.

### Morphological alterations in HUVEC upon incubation with DMOG

**Figure 7:**
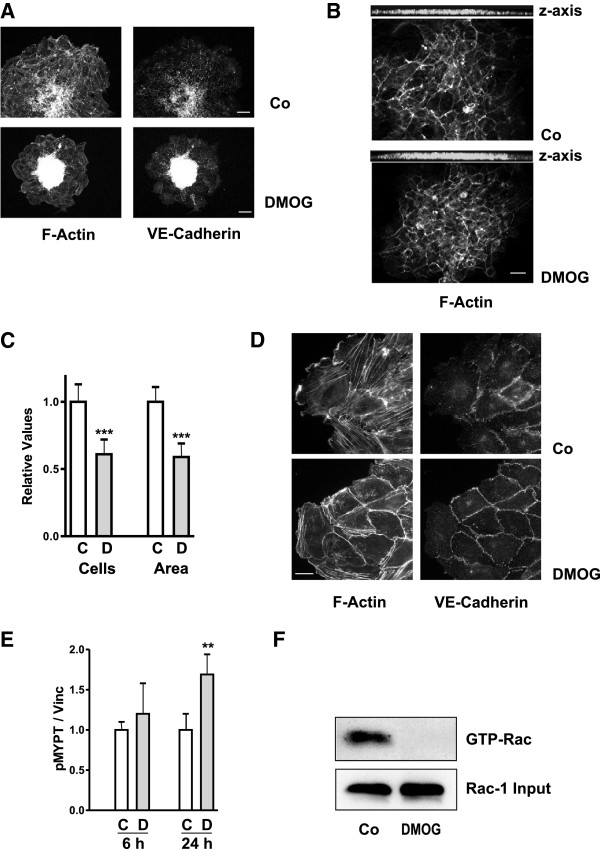
**HUVEC are sensitive to DMOG. (A)** HUVEC were organized into spheroids and plated on collagen IV-coated plates in the presence or absence of 1 mM DMOG. F-actin and VE-cadherin were visualized by PromoFluor phalloidin staining and indirect immunofluorescence, respectively. Scale bar: 20 μm. **(B)** F-actin structures within the spheroids were visualized by apotome technique. Scale bar: 20 μm. Images were merged to show the height of the spheroids in z-axis. **(D)** Higher magnifications of F-actin fibers and VE-cadherin structures as shown in (Figure 7A). Scale bar: 20 μm. **(C)** After 24 h, nuclei were stained with Hoechst and the number of migrated cells was counted using ImageJ. Cell numbers were quantified in 35 spheroids of 5 different preparations. The graph summarizes the data of 20 spheroids of three different isolates (means ± SD). The area covered by F-actin stained cells (means ± SD) was determined in 35 spheroids of 5 different isolates. In each experiment the mean value of control spheroids was set to 1, error bars reflecting the variability within one experiment . *** p < 0.0001, Student’s *t*-test. **(E)** HUVEC were cultured in cell culture dishes treated with DMOG (1 mM) for 6 and 24 h. Phosphorylated MYPT was detected by Western blotting. Data are means ± SD of 3 experiments performed in duplicate. In each experiment the mean value of controls was set to 1, error bars of controls reflecting variability of biological samples. ** p < 0.01, Student’s *t*-test. **(F)** HUVEC cultured in cell culture dishes were treated with DMOG (1 mM) for 24 h. GTP-Rac was precipitated from 700 μg cellular protein. Total Rac was detected in 25 μg cellular protein. The blot is representative of 2 precipitations.

Effects of DMOG were further investigated in human primary umbilical vein endothelial cells (HUVEC), which were organized into spheroids and were then treated with DMOG. Control cells migrated off the spheroids which flattened and lost their structure, whereas spheroids remained organized in the presence of DMOG (Figure 7A/B). Representative overviews of F-actin-stained cells are shown in Figure 7A. A more detailed view of the structure within the spheroids was obtained by apotome technique (Figure 7B). Merged images projected on the z-axis revealed the different height of the spheroids, which was obvious even after fixation and staining. As observed with microvascular cells, the number of cells which migrated off the spheroids was significantly decreased upon treatment with DMOG (Figure 7C). Migration of these cells was impaired as the area covered was much smaller than in controls, i.e. the migration distance of individual cells was reduced (Figure 7C). DMOG also altered the cytoskeletal organization of HUVEC. Migrating control cells were characterized by distinct F-actin fibers at the front of lamellipodia and cell spanning F-actin fibers (Figure 7D). In contrast, DMOG-treated cells lacked extended lamellipodia, F-actin fibers were concentrated subcortical with very little cell spanning fibers (Figure 7D). Reorganization was also reflected by VE-cadherin distribution: VE-caderin was poorly visible in the periphery of migrating control cells (Figure 7A) due to the loose perpendicular structures observed at higher magnification (Figure 7D). The tight cell-cell contacts in DMOG-treated cells correlated with VE-cadherin organized as distinct band along the cell boundaries (Figure 7D). In contrast to microvascular cells we observed increased levels of pMYPT upon incubation with DMOG (Figure 7E). However, in line with the phenotypic alterations and the results obtained in glEND.2 cells, Rac-1 activity was strongly reduced in HUVEC exposed to DMOG (Figure 7F). Reduction of Rac-1 activity thus increased cell-cell interactions in both, glEND.2 cells and HUVEC, whereas cell matrix interactions were modulated differentially in both cell types, leading to more directional migration in glEND.2 cells and reduced migration in HUVEC.

## Discussion

**Figure 8:**
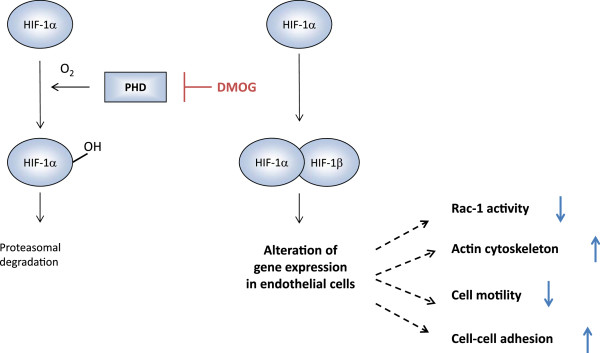
**Schematic summary of DMOG-induced modulation of endothelial cells morphology.** Incubation of the cells with DMOG prevents PHD-dependent degradation of HIF-1α. Stabilized HIF-1α modulates gene expression, which, as shown in our studies, leads to reduced Rac-1 activity associated with alterations in cell morphology.

In this study we show that inhibition of HIF prolylhydroxylases (PHDs) by DMOG stabilizes HIF-1α, which resulted in reduced Rac-1 activity and markedly altered F-actin structures. These alterations led to sustained cell-cell interaction and reduced cell motility in endothelial cells, microvascular glEND.2 cells and HUVEC (summarized in Figure 8).

As sensors of oxygen tension, PHDs are important regulators of various steps of angiogenesis. Most of their effects can be attributed to their role in regulating the stability of HIF transcription factors [5,34]. PHD inhibitors such as DMOG stabilize both HIF-α isoforms, but also activate HIF-independent pathways, for example NF-kB signaling [9,34,35]. Using stably transfected glEND.2 cells, our data clearly showed that HIF-1α was the main mediator of the observed morphological alterations and necessary for the underlying inhibition of Rac-1/PAK signaling after inhibition of PHDs. These findings were further confirmed by transient inhibition of HIF-1α using siRNA, which also prevented DMOG-mediated downregulation of phosphorylated PAK. Interestingly, spheroids formed by cells in which HIF-2α was downregulated by shRNA were consistently smaller than those obtained from shHIF-1α or shGFP clones. Moreover, these cells were less tightly attached as shown upon processing for F-actin staining, where they tended to roll away (see Figure 3D). A tendency to reduced matrix adhesion was also reported in immortalized endothelial cells obtained from lungs of HIF-2α knockout mice [36]. These results suggest distinct roles for HIF-1α and HIF-2α in microvascular endothelial cell adhesion, and especially the effect of HIF-2α warrants further investigation.

Incubation of spheroids with the PHD inhibitor DMOG affected both, cells in three dimensional clusters and migrating cells. Our *in vitro* model addressed two aspects of endothelial cell interaction: homotypic cell-cell interactions which prevailed within the spheroids and determined the size of the three dimensional spheroids, as well as cell-matrix adhesions which were essential for cell spreading and movement of the cells on the plates. These aspects of spheroid migration are not independent, but potentially interrelated: strong cell-cell interactions would be expected to prevent migration on extracellular matrices, whereas loosening of cell-cell contacts would favor movement of the cells out of the spheroid. With regard to molecular mechanisms related to these processes, we previously reported reduced spheroid size and increased numbers of migrating endothelial cells upon inhibition of Rho kinases which altered cytoskeletal structures and gene expression [19]. By contrast, stabilization of HIF-1α was associated with an inhibition of Rac-1 activity and an increased spheroid size indicative of enhanced cell-cell adhesion. In HUVEC, DMOG not only increased adhesion within the spheroids, but also in migrating cells associated with a significant reduction in cell migration.

In the model system used here, the driving forces for cell migration were the differences in adhesive strength between cells within the spheroids and cell-matrix interactions on the matrix-coated cover slips. Attachment of the cells to the extracellular matrix, either collagen IV or fibronectin, was stronger than cell-cell adhesion between neighboring cells within spheroids. In this experimental setting, microvascular cells migrated readily, whereas they were barely mobile when firmly attached to the substratum, i.e. in scratch wounding assays [19]. DMOG induced strong F-actin fibers in the migrating microvascular glEND.2 cells. The alteration of F-actin stress fibers was observed primarily in migrating cells, not in cells imbedded in a monolayer or within the spheroids. This suggests that structural effects of PHD inhibitors will be most prominent in the context of neovascularization, with lesser effects on cells in intact vessels. Notably, as the endothelial cells needed serum for survival, migrating and adherent cells were exposed to the same soluble mediators, and were not activated by single stimuli. This model system thus differs from other studies which analyzed short term effects of angiogenic factors such as thrombin or VEGF on endothelial cells in confluent monolayers (summarized in [37]). Hypoxia-mediated transient alterations in the F-actin cytoskeleton and a redistribution of vimentin filaments have been reported in pulmonary endothelial cells to occur within one hour [38]. In our experiments, more than 3 h were necessary to induce sustained morphological alterations, even though HIF-1α was induced rapidly within 1 h in glEND.2 cells [29]. Within this time frame, no changes in F-actin structures were detectable upon DMOG treatment. This suggested that changes were driven by HIF-1α-dependent alterations in gene expression rather than by rapid interactions between proteins. Stabilization of HIF-1α transcription factors by PHD inhibitors leads to a whole set of changes in gene expression which mostly overlaps with those induced by the exposure of cells to hypoxia [39].

Rho and Rac GTPases are interacting regulators of the organization and dynamics of the actin cytoskeleton [23,37]. Our data indicated that DMOG-mediated alterations in cell migration and cytoskeletal remodeling were primarily due to reduced Rac-1 signaling. In line with our observations, Pankov et al. had previously described that decreased Rac-1 activity switched cell migration patterns of fibroblasts from random to directionally persistent migration, a phenotype which was not observed upon reduction of RhoA or Cdc42 activity [40]. Several lines of evidence indicated that Rac-1 signaling was reduced downstream of HIF-1α: (a) stabilization of F-actin fibers and increased residual spheroid size was observed in control cells, but not in shHIF-1α cell clones; (b) DMOG-mediated reduction of PAK activity was less pronounced in shHIF-1α cells and (c), inhibition of Rac-1 activity affected spheroid size also in shHIF-1α cells. Long-term stabilization of HIF-1α by inhibition of PHDs, which also mimics chronic hypoxia, thus reduces Rac-1 signaling in endothelial cells. This is in contrast to its role in short-term hypoxia, where Rac-1 was reported to act upstream of HIF-1α and was required for the induction of HIF-1α protein expression and transcriptional activity [41]. Physiologically, our observations are reminiscent of the *in vivo* piglet model of hypoxia-induced neonatal pulmonary hypertension. Lung endothelial cells obtained from these animals also showed reduced Rac-1 activity and increased F-actin stress fibers [23].

As shown in detailed studies, Rho GTPases are implicated in a complex network of regulatory proteins the interaction of which is dependent on the cellular context and the mode of activation or inhibition [42-44]. As obvious examples for this diversity we observed different regulation of MYPT phosphorylation in glEND.2 cells and HUVEC with reduction of MYPT phosphorylation in glEND.2 cells and a slight increase in HUVEC. Furthermore, both cell types also differed in their arrangement of F-actin fibers, being primarily cortical in DMOG-treated HUVEC and cell spanning in glEND.2 cells. More detailed studies are warranted to dissect the spatio-temporal regulation of actin-modulating proteins and their impact on cell motility in both cell types.

Our model system not only addressed effects of PHD inhibition on migrating endothelial cells, but also on endothelial cells organized in three dimensional spheroids. DMOG increased spheroid size in a HIF-1α-dependent fashion, indicating strengthened cell-cell contacts as also shown by strong VE-cadherin lining at the cell borders. As shown by different pharmacological inhibitors, reduced Rac-1 activity promoted stabilization of spheroids. These data seem to be in contrast to other reports which show that activation of Rac-1 is needed to promote stability of endothelial barriers [22]. The discrepancy may be due to substantial differences in experimental design. As outlined above, inhibition of PHDs did not reduce the cellular content of Rac-1 proteins as seen in cells transfected with dominant negative Rac-1 [45,46]. Furthermore, inhibition of Rac-1 activity occurred over an extended time and thus differed from rapid and more drastic alterations studied in other model systems. Comparison of DMOG and the inhibitors of Rac-1 activity, EHT1864 and NSC2367, showed more pronounced effects of DMOG compared to the inhibitors. This indicated that the stabilizing effect of DMOG was not limited to the inhibition of Rac-1 activity, but involved additional molecular mediators, which remain to be elucidated. Stabilization of endothelial cell-cell interactions was in line with *in vivo* data obtained by Mazzone et al. [7]. They observed normalization of tumor vessel formation in PHD2 +/− mice, a model system with only a partial reduction in PHD activity. Tumor vessel normalization was related to less tumor invasiveness and less metastasis [47], consistent with stabilization of endothelial cell interactions. In summary our data provides evidence that pharmacological inhibition of PHDs alters cell-cell adhesion and cell motility in endothelial cells through HIF-1α- and Rac-1-dependent pathways.

## Conclusions

PHD inhibitors provide a novel therapeutic opportunity to reduce ischemia-reperfusion injury in various organs [48,49]. The beneficial effects include amelioration of blood flow [17,50] and reduction of ischemic damage, which cannot be attributed to single cell types but have to involve different cellular compartments including endothelial, parenchymal and immune cells. Our *in vitro* data provide the first evidence that PHD inhibitors support vascular endothelial cell stability through HIF-1α dependent cytoskeletal reorganizations and thus may contribute to vessel normalization and improved blood supply.

## Methods

### Cell culture

The murine glomerular microvascular endothelial cell line (glEND.2) was kindly provided by R. Hallmann (Muenster, Germany). Cells were characterized by positive staining for typical endothelial cell markers MECA-32 and CD-31, and the lack of staining of mesangial cell markers such as α-smooth muscle actin and α8-integrin, as well as epithelial cell markers such as WT-1 and cytokeratin [51]. Cells were cultured at 37°C and 7.5% CO_2_ in Dulbecco’s modified Eagle’s medium (DMEM) containing 10% FCS and routinely split in a 1:5 ratio as described previously [29]. Cells were cultured under hypoxic conditions in an incubator with 1% O_2_, 5% CO_2_ and balance nitrogen. Human umbilical vein endothelial cells (HUVEC) were isolated and cultured as described [52]. Isolation of human cells was approved by the local ethics committee (approval number #3759) and written consent was obtained from all donors.

### Generation of stable HIF-1α and HIF-2α knockdown glEND.2 cells

glEND.2 cells were stably transfected with shRNA producing constructs by lentiviral infection. Lentiviral particles were produced by transfecting HEK 293 T packaging cells (ATCC) simultaneously with Pax2 plasmid, PMD2 plasmid (both Addgene) and shRNA containing plasmid (Open Biosystems) using FuGene transfection reagent (Roche) in OptiMEM medium (Life Technologies). 24 h after transfection, supernatant containing lentiviral particles were collected, filtered through a 45 μm syringe filter and added directly to subconfluent glEND.2 cells. After 24 h, cells were washed and cultured with selection medium containing puromycin (Sigma) at a final concentration of 2.5 μg/ml. Knockdown efficiency was controlled by Western blot analysis of HIF-α protein expression and by quantitative real-time PCR of classical HIF-α target genes such as phosphoglycerate kinase (PGK) and glucose transporter 1 (GLUT-1).

### Migration assays

Cell spheroids (400 cells per spheroid) were created using the hanging drop method [29,53]. In brief, cells were suspended in DMEM with 0.24% methylcellulose. Cell suspension drops (400 cells/25 μl) were deposited onto the underside of the lid of a tissue culture dish. The lid was inverted and incubated overnight at 37°C.

For the spheroid migration assay, single spheroids were deposited separately on fibronectin-coated glass plates and stimulated as described in the Results section. F-actin and nuclei were stained for quantification purposes to determine area of residual spheroids, area and cell number of migrated spheroids of at least 5 spheroids per experimental group using ImageJ software. Live cell imaging was performed as described [19].

### Western blot analysis

Western blot analyses were performed by standard procedures as described previously [19]. Cells were cultured as monolayers in cell culture dishes. To detect phospho-PAK or phospho-MYPT, cells were lysed in SDS containing buffer. For HIF-α, cell lysates were prepared in a urea containing buffer [54]. To detect HIF-1α 30 μg of total protein was loaded. Nuclear extracts of glEND.2 clones were prepared as described [6] and 20 μg protein analyzed for HIF-2α expression.

The following antibodies were used: mouse anti-vinculin (SC-5573), mouse anti-RhoA (SC-418) and peroxidase-conjugated donkey anti-goat IgG (SC-2020), goat anti-rat IgG (SC-2006) from Santa Cruz; mouse anti-Rac-1 (610651) from BD Transduction Laboratories; rabbit anti-phospho-PAK (2601) and rabbit anti-phospho-MYPT (Thr853) (#4563) from Cell Signaling, rabbit anti-HIF-1α (NB100-449, Novus Biologicals), goat anti-HIF-2α (AF2997, R&D Systems), rat anti-VE-cadherin (14–1441, eBiosciences), mouse anti β–actin; sheep anti-mouse IgG (NA931V), anti-rat donkey anti-rabbit IgG (NA934V) from Amersham Biosciences.

Immunoreactive proteins were visualized by the enhanced chemiluminescence detection system (ECL-Plus, Amersham). Immunoreactive bands were quantified using the luminescent image analyzer (LAS-1000 Image Analyzer, Fujifilm) and AIDA 4.15 image analyzer software (Raytest). To correct for equal loading and blotting, all blots were redetected with antibodies directed against vinculin or β-actin. For quantification purposes, the ratio of the specific protein band and a control protein was calculated.

### Quantitative Real-Time PCR

Total RNA was isolated from glEND.2 cells after incubation with 1% oxygen for 18 h using RNeasy kit (Qiagen) according to the manufacturer’s protocol. First-strand synthesis was performed with 1 μg of total RNA with the MaximaTM First Strand cDNA synthesis kit (Fermentas) according to the manufacturer’s recommendations. cDNAs were amplified in SYBR Green MaximaTM Master Mix (Fermentas) with a StepOne PlusTM Real Time-PCR system (Applied Biosystems). Expression levels were related to 18 s using the delta-ct method. Primer sequences are as follows: Glut-1: 5′-ACGAGGAGCACCGTGAAGAT and 5′-GGGCATGTGCTTCCAGTATGT; PGK: 5′-CAAATTTGATGAGAATGCCAAGACT and 5′-TTCTTGCTGCTCTCAGTACCACA; 18S 5′-CGGACAGGATTGACAGATTG and 5′-CAAATCGCTCCACCAACTAA.

### Immunocytochemistry

Immunocytochemistry of glEND.2 cells was performed essentially as described [19]. Primary antibodies were those used for Western blotting. Secondary antibodies (1:500, PromoFluor Fluor® 488 anti-mouse A21202 or 488 anti-rat A11006) were from Molecular Probes. F-actin was stained with PromoFluor 488 or 555 phalloidin from PromoKine, nuclei were visualized with Hoechst (Sigma-Aldrich). After mounting, slides were viewed using a Nikon fluorescence microscope. Digital images were recorded using Spot imaging software (Diagnostic Instruments). Co-localization of proteins was confirmed by confocal microscopy using a Zeiss LSM 710 scanning unit equipped with an Argon laser, a HeNe 633 laser and a DPSS 561–10 laser on an Axio Observer Z1 inverted microscope. To avoid spectral crosstalk between the used fluorochromes and to sustain high sensitivity scanning was performed in two sequential scanning steps.

All stainings shown are representative of at least 3 independent experiments. ImageJ software was used to quantify cell numbers and areas covered by spheroids or cells.

### Transfection - siRNA Transfection

To down-regulate HIF-1α expression, endothelial cells (glEND.2) were transfected with the specific siRNAs (50 nM) 3 h after seeding using HiPerFect (QIAGEN) according to the manufacturer’s instructions. Specific siRNAs directed against HIF-1α (sense 5′-GCC-ACU-UCG-AAG-UAG-UGC-U), or an irrelevant siRNA (5′-GGA-UGG-CAU-CUC-GGA-GCU-C) were obtained from Eurogentec. Experiments were performed 48 h after transfection.

### DNA transfection

Cells were seeded on collagen IV-coated cover slips at low density (12,500 cells/cm^2^). The next day, cDNA constructs encoding constitutively active Rac-1 (human pEGFP/Rac1(V12)) or RhoA (human pEGFP/RhoA(V14)) were transfected using X-treme HD (Roche) following the manufacturer’s instructions.

### Determination of Rac-1 activity

Rac-1 activity was determined essentially as described previously [55]. The GTP-bound form of Rac-1 was recovered from 500 μg of cell lysate by affinity precipitation using a GST-fusion protein carrying the Rac-1 binding domain of PAK1B as an activation-specific probe for endogenous Rac-1 [56].

### Data analysis

Data are presented as means ± s.d or s.e.m. of n > 3 independent experiments as detailed in the legends. To compare two samples, Student’s *t*-test or one sample *t*-test was used. ANOVA with Tukey Kramer multiple comparison or Dunnett’s post hoc test was used to compare multiple measurements (Prism GraphPad Software, La Jolla, CA, USA). A p value < 0.05 was considered significant.

### Materials

Cell culture materials were purchased from PAA Laboratories. The following biochemicals were used: H1152, (*S*)-(+)-2-methyl-1-[(4-methyl-5-isoquinolinyl)sulfonyl]-homopiperazine, (Alexis Biochemicals), collagen-IV (Sigma), fibronectin (BD Bioscience); methylcellulose 4000 (Fluka); dimethyloxalyl glycine (DMOG, Cayman Chemical). Two Rac-1 inhibitors were used: EHT1864 (Tocris), which places Rac-1 in an inert and inactive state [32] and NSC23677 (Calbiochem), which specifically inhibits Rac-1 GDP/GTP exchange activity [31].

## Abbreviations

HIF: Hypoxia-inducible transcription factor; GLUT-1: Glucose transporter 1; HUVEC: Human umbilical vein endothelial cell; MYPT: Myosin phosphatase target protein; PAK: p21-activated kinase; PGK: Phosphoglycerate kinase; PHD: Prolyl hydroxylase domain enzyme; ROCK: Rho kinase, small G protein Rho-associated kinase; VEGF: Vascular endothelial growth factor; VHL: von Hippel-Lindau; DMOG: dimethyloxalyl glycine.

## Competing interests

The authors declare no competing interests.

## Authors’ contributions

AW generated and characterized the stably transfected glEND.2 clones and drafted the manuscript; JB and MR performed experiments; KUE drafted the manuscript; CD performed confocal microscopy; IC did experiments with HUVECs; KG provided reagents for overexpression and analysis of GTPases, MGS designed the study, evaluated the data and wrote the manuscript; all authors critically read and approved the manuscript.

## Supplementary Material

Additional file 1 **Live cell imaging of glEND.2 cells.** Spheroids of glEND.2 cells were plated on collagen IV-coated glass plates and treated with or without DMOG (1 mM). Movement of cells was monitored with an inverted microscope (Leica DMI3000B) in phase contrast mode using a 20x 0.4 NA objective with a 0.5x video coupler. The microscope was equipped with an incubation chamber to maintain the spheroids at 37°C and 7.5% CO2. 200 images were taken every 5 min for 17 h. Click here for file

Additional file 2 **Figure S2: DMOG-mediated structural alterations in low-density glEND.2.** (A) glEND.2 cells were seeded at low density on fibronectin-coated glass plates and incubated for 24 h with or without 1 mM DMOG. Cells were visualized by staining F-actin with PromoFluor phalloidin and staining of nuclei with Hoechst. In each condition, 6 visual fields were randomly chosen, the number of cells was counted and the area covered by the cells determined using ImageJ software. The graph depicts area per cell in arbitrary units, means + SD of 6 fields. ** p < 0.01, Student’s *t*-test. (B) glEND.2 cells were seeded at low density on fibronectin–coated glass plates and incubated for 24 h with or without 1 mM DMOG. F-actin fibers were visualized by PromoFluor phalloidin staining. DMOG-treated cells showed strong F-actin fibers and appeared more spread. Scale bar: 20 μm. (C) glEND.2 cells were seeded to form a confluent monolayer and then incubated with DMOG for 24 h. F-actin fibers were visualized by PromoFluor phalloidin staining. Scale bar: 20 μm. Click here for file

Additional file 3 **Figure S3: Generation of spheroids in the presence of DMOG.** (A) Spheroids were generated by the hanging drop method in the presence or absence of DMOG. For quantification, spheroids were allowed to adhere on plates for 3 h. Spheroids with DMOG appeared more tightly packed forming round structures compared to the control spheroids which appeared less organized. Accordingly, the area covered by DMOG-treated spheroids was reduced. The graph summarizes data (means + SD) of 30 spheroids of three different experiments. In each experiment the mean value of control spheroids was set to 1, error bars reflect the variability within one experiment. *** p < 0.001, Student’s *t*-test. (B) After fixation the nuclei were stained with DAPI to assess the height of the spheroids by apotome technique. Layers were merged and the height of the spheroid approximated by the fluorescence of the z-axis. Data of 6 spheroids each of one representative experiment are depicted. *** p < 0.001, Student’s *t*-test. Click here for file

Additional file 4 **Figure S4: Inhibition of Rac-1 is effective independently of HIF.** Spheroids of shHIF-1 and shHIF-2 clones were treated with 100 μM NSC23677 overnight. The area covered by residual spheroids was determined in 6 spheroids for each condition. Mean value of control cells was set to 1. * p < 0.05, Student’s *t*-test. The Rac-1 inhibitor increased spheroid size independently of HIF-1 or HIF-2 knockdown. Scale bar: 20 μm. Click here for file

## References

[B1] Rey S Semenza GL Hypoxia-inducible factor-1-dependent mechanisms of vascularization and vascular remodelling Cardiovasc Res 2010 11 236 242 10.1093/cvr/cvq045 20164116 PMC2856192

[B2] Pugh CW Ratcliffe PJ Regulation of angiogenesis by hypoxia: role of the HIF system Nat Med 2003 11 677 684 10.1038/nm0603-677 12778166

[B3] Greer SN Metcalf JL Wang Y Ohh M The updated biology of hypoxia-inducible factor EMBO J 2012 11 2448 2460 10.1038/emboj.2012.125 22562152 PMC3365421

[B4] Weidemann A Johnson RS Biology of HIF-1alpha Cell Death Differ 2008 11 621 627 10.1038/cdd.2008.12 18259201

[B5] Fong GH Takeda K Role and regulation of prolyl hydroxylase domain proteins Cell Death Differ 2008 11 635 641 10.1038/cdd.2008.10 18259202

[B6] Weidemann A Kerdiles YM Knaup KX Rafie CA Boutin AT Stockmann C Takeda N Scadeng M Shih AY Haase VH The glial cell response is an essential component of hypoxia-induced erythropoiesis in mice J Clin Invest 2009 11 3373 3383 19809162 10.1172/JCI39378 PMC2769183

[B7] Mazzone M Dettori D Leite De Oliveira R Loges S Schmidt T Jonckx B Tian YM Lanahan AA Pollard P Ruiz De Almodovar C Heterozygous deficiency of PHD2 restores tumor oxygenation and inhibits metastasis via endothelial normalization Cell 2009 11 839 851 10.1016/j.cell.2009.01.020 19217150 PMC4037868

[B8] Jokilehto T Jaakkola PM The role of HIF prolyl hydroxylases in tumour growth J Cell Mol Med 2010 11 758 770 10.1111/j.1582-4934.2010.01030.x 20178464 PMC3823110

[B9] Chan DA Kawahara TL Sutphin PD Chang HY Chi JT Giaccia AJ Tumor vasculature is regulated by PHD2-mediated angiogenesis and bone marrow-derived cell recruitment Cancer Cell 2009 11 527 538 10.1016/j.ccr.2009.04.010 19477431 PMC2846696

[B10] Hulten LM Levin M The role of hypoxia in atherosclerosis Curr Opin Lipidol 2009 11 409 414 10.1097/MOL.0b013e3283307be8 19644366

[B11] Andrikopoulou E Zhang X Sebastian R Marti G Liu L Milner SM Harmon JW Current Insights into the role of HIF-1 in cutaneous wound healing Curr Mol Med 2011 11 218 235 10.2174/156652411795243414 21375491

[B12] Senbanerjee S Thirunavukkarasu M Rishi MT Sanchez JA Maulik N Maulik G HIF-Prolyl hydroxylases and cardiovascular diseases Toxicol Mech Methods 2012 11 347 358 10.3109/15376516.2012.673088 22424133

[B13] Kasivisvanathan V Shalhoub J Lim CS Shepherd AC Thapar A Davies AH Hypoxia-inducible factor-1 in arterial disease: a putative therapeutic target Curr Vasc Pharmacol 2011 11 333 349 10.2174/157016111795495602 20807188

[B14] Nordgren IK Tavassoli A Targeting tumour angiogenesis with small molecule inhibitors of hypoxia inducible factor Chem Soc Rev 2011 11 4307 4317 10.1039/c1cs15032d 21483947

[B15] Shen X Wan C Ramaswamy G Mavalli M Wang Y Duvall CL Deng LF Guldberg RE Eberhart A Clemens TL Gilbert SR Prolyl hydroxylase inhibitors increase neoangiogenesis and callus formation following femur fracture in mice J Orthop Res 2009 11 1298 1305 10.1002/jor.20886 19338032 PMC3767389

[B16] Bao W Qin P Needle S Erickson-Miller CL Duffy KJ Ariazi JL Zhao S Olzinski AR Behm DJ Pipes GC Chronic inhibition of hypoxia-inducible factor prolyl 4-hydroxylase improves ventricular performance, remodeling, and vascularity after myocardial infarction in the rat J Cardiovasc Pharmacol 2010 11 147 155 10.1097/FJC.0b013e3181e2bfef 20714241

[B17] Ogle ME Gu X Espinera AR Wei L Inhibition of prolyl hydroxylases by dimethyloxaloylglycine after stroke reduces ischemic brain injury and requires hypoxia inducible factor-1alpha Neurobiol Dis 2012 11 733 742 10.1016/j.nbd.2011.10.020 22061780 PMC3286647

[B18] Kroll J Epting D Kern K Dietz CT Feng Y Hammes HP Wieland T Augustin HG Inhibition of Rho-dependent kinases ROCK I/II activates VEGF-driven retinal neovascularization and sprouting angiogenesis Am J Physiol Heart Circ Physiol 2009 11 H893 H899 10.1152/ajpheart.01038.2008 19181962

[B19] Breyer J Samarin J Rehm M Lautscham L Fabry B Goppelt-Struebe M Inhibition of Rho kinases increases directional motility of microvascular endothelial cells Biochem Pharmacol 2012 11 616 626 10.1016/j.bcp.2011.12.012 22192821

[B20] Warnecke C Griethe W Weidemann A Jurgensen JS Willam C Bachmann S Ivashchenko Y Wagner I Frei U Wiesener M Eckardt KU Activation of the hypoxia-inducible factor-pathway and stimulation of angiogenesis by application of prolyl hydroxylase inhibitors FASEB J 2003 11 1186 1188 12709400 10.1096/fj.02-1062fje

[B21] Prasain N Stevens T The actin cytoskeleton in endothelial cell phenotypes Microvasc Res 2009 11 53 63 10.1016/j.mvr.2008.09.012 19028505 PMC2700738

[B22] Spindler V Schlegel N Waschke J Role of GTPases in control of microvascular permeability Cardiovasc Res 2010 11 243 253 10.1093/cvr/cvq086 20299335

[B23] Wojciak-Stothard B Tsang LY Paleolog E Hall SM Haworth SG Rac1 and RhoA as regulators of endothelial phenotype and barrier function in hypoxia-induced neonatal pulmonary hypertension Am J Physiol Lung Cell Mol Physiol 2006 11 L1173 L1182 10.1152/ajplung.00309.2005 16428270

[B24] Zhang XZ Huang X Qiao JH Zhang JJ Zhang MX Inhibition of hypoxia-induced retinal neovascularization in mice with short hairpin RNA targeting Rac1, possibly via blockading redox signaling Exp Eye Res 2011 11 473 481 10.1016/j.exer.2011.03.005 21414312

[B25] Xue Y Li NL Yang JY Chen Y Yang LL Liu WC Phosphatidylinositol 3′-kinase signaling pathway is essential for Rac1-induced hypoxia-inducible factor-1(alpha) and vascular endothelial growth factor expression Am J Physiol Heart Circ Physiol 2011 11 H2169 H2176 10.1152/ajpheart.00970.2010 21357506

[B26] Diebold I Djordjevic T Hess J Gorlach A Rac-1 promotes pulmonary artery smooth muscle cell proliferation by upregulation of plasminogen activator inhibitor-1: role of NFkappaB-dependent hypoxia-inducible factor-1alpha transcription Thromb Haemost 2008 11 1021 1028 19132225

[B27] Kayyali US Pennella CM Trujillo C Villa O Gaestel M Hassoun PM Cytoskeletal changes in hypoxic pulmonary endothelial cells are dependent on MAPK-activated protein kinase MK2 J Biol Chem 2002 11 42596 42602 10.1074/jbc.M205863200 12202485

[B28] Partridge CA Hypoxia and reoxygenation stimulate biphasic changes in endothelial monolayer permeability Am J Physiol 1995 11 L52 L58 7631814 10.1152/ajplung.1995.269.1.L52

[B29] Samarin J Wessel J Cicha I Kroening S Warnecke C Goppelt-Struebe M FoxO proteins mediate hypoxic induction of connective tissue growth factor in endothelial cells J Biol Chem 2010 11 4328 4336 10.1074/jbc.M109.049650 20018872 PMC2836037

[B30] Patel SA Simon MC Biology of hypoxia-inducible factor-2alpha in development and disease Cell Death Differ 2008 11 628 634 10.1038/cdd.2008.17 18259197 PMC2882207

[B31] Gao Y Dickerson JB Guo F Zheng J Zheng Y Rational design and characterization of a Rac GTPase-specific small molecule inhibitor Proc Natl Acad Sci USA 2004 11 7618 7623 10.1073/pnas.0307512101 15128949 PMC419655

[B32] Shutes A Onesto C Picard V Leblond B Schweighoffer F Der CJ Specificity and mechanism of action of EHT 1864, a novel small molecule inhibitor of Rac family small GTPases J Biol Chem 2007 11 35666 35678 10.1074/jbc.M703571200 17932039

[B33] Galan Moya EM Le Guelte A Gavard J PAKing up to the endothelium Cell Signal 2009 11 1727 1737 10.1016/j.cellsig.2009.08.006 19720142

[B34] Coulon C Georgiadou M Roncal C De Bock K Langenberg T Carmeliet P From vessel sprouting to normalization: role of the prolyl hydroxylase domain protein/hypoxia-inducible factor oxygen-sensing machinery Arterioscler Thromb Vasc Biol 2010 11 2331 2336 10.1161/ATVBAHA.110.214106 20966400

[B35] Takeda K Ichiki T Narabayashi E Inanaga K Miyazaki R Hashimoto T Matsuura H Ikeda J Miyata T Sunagawa K Inhibition of prolyl hydroxylase domain-containing protein suppressed lipopolysaccharide-induced TNF-alpha expression Arterioscler Thromb Vasc Biol 2009 11 2132 2137 10.1161/ATVBAHA.109.196071 19762779

[B36] Skuli N Liu L Runge A Wang T Yuan L Patel S Iruela-Arispe L Simon MC Keith B Endothelial deletion of hypoxia-inducible factor-2alpha (HIF-2alpha) alters vascular function and tumor angiogenesis Blood 2009 11 469 477 10.1182/blood-2008-12-193581 19439736 PMC2714217

[B37] Bryan BA D'Amore PA What tangled webs they weave: Rho-GTPase control of angiogenesis Cell Mol Life Sci 2007 11 2053 2065 10.1007/s00018-007-7008-z 17530172 PMC11138424

[B38] Liu T Guevara OE Warburton RR Hill NS Gaestel M Kayyali US Regulation of vimentin intermediate filaments in endothelial cells by hypoxia Am J Physiol Cell Physiol 2010 11 C363 C373 10.1152/ajpcell.00057.2010 20427712 PMC2928624

[B39] Elvidge GP Glenny L Appelhoff RJ Ratcliffe PJ Ragoussis J Gleadle JM Concordant regulation of gene expression by hypoxia and 2-oxoglutarate-dependent dioxygenase inhibition: the role of HIF-1alpha, HIF-2alpha, and other pathways J Biol Chem 2006 11 15215 15226 10.1074/jbc.M511408200 16565084

[B40] Pankov R Endo Y Even-Ram S Araki M Clark K Cukierman E Matsumoto K Yamada KM A Rac switch regulates random versus directionally persistent cell migration J Cell Biol 2005 11 793 802 10.1083/jcb.200503152 16129786 PMC2171343

[B41] Hirota K Semenza GL Rac1 activity is required for the activation of hypoxia-inducible factor 1 J Biol Chem 2001 11 21166 21172 10.1074/jbc.M100677200 11283021

[B42] Beckers CM van Hinsbergh VW van Nieuw Amerongen GP Driving Rho GTPase activity in endothelial cells regulates barrier integrity Thromb Haemost 2010 11 40 55 20062930 10.1160/TH09-06-0403

[B43] Szulcek R Beckers CM Hodzic J de Wit J Chen Z Grob T Musters RJ Minshall RD Van Hinsbergh VW van Nieuw Amerongen GP Localized RhoA GTPase activity regulates dynamics of endothelial monolayer integrity Cardiovasc Res 2013 11 471 482 10.1093/cvr/cvt075 23536606 PMC3841417

[B44] Menke A Giehl K Regulation of adherens junctions by Rho GTPases and p120-catenin Arch Biochem Biophys 2012 11 48 55 10.1016/j.abb.2012.04.019 22583808

[B45] Tan W Palmby TR Gavard J Amornphimoltham P Zheng Y Gutkind JS An essential role for Rac1 in endothelial cell function and vascular development FASEB J 2008 11 1829 1838 10.1096/fj.07-096438 18245172

[B46] Waschke J Baumgartner W Adamson RH Zeng M Aktories K Barth H Wilde C Curry FE Drenckhahn D Requirement of Rac activity for maintenance of capillary endothelial barrier properties Am J Physiol Heart Circ Physiol 2004 11 H394 H401 14512275 10.1152/ajpheart.00221.2003

[B47] De Bock K De Smet F Leite De Oliveira R Anthonis K Carmeliet P Endothelial oxygen sensors regulate tumor vessel abnormalization by instructing phalanx endothelial cells J Mol Med (Berl) 2009 11 561 569 10.1007/s00109-009-0482-z 19455291

[B48] Bernhardt WM Wiesener MS Scigalla P Chou J Schmieder RE Gunzler V Eckardt KU Inhibition of prolyl hydroxylases increases erythropoietin production in ESRD J Am Soc Nephrol 2010 11 2151 2156 10.1681/ASN.2010010116 21115615 PMC3014028

[B49] Ong SG Hausenloy DJ Hypoxia-inducible factor as a therapeutic target for cardioprotection Pharmacol Ther 2012 11 69 81 10.1016/j.pharmthera.2012.07.005 22800800

[B50] Deng A Arndt MA Satriano J Singh P Rieg T Thomson S Tang T Blantz RC Renal protection in chronic kidney disease: hypoxia-inducible factor activation vs. angiotensin II blockade Am J Physiol Renal Physiol 2010 11 F1365 F1373 10.1152/ajprenal.00153.2010 20881034 PMC3006314

[B51] Li ZD Bork JP Krueger B Patsenker E Schulze-Krebs A Hahn EG Schuppan D VEGF induces proliferation, migration, and TGF-beta1 expression in mouse glomerular endothelial cells via mitogen-activated protein kinase and phosphatidylinositol 3-kinase Biochem Biophys Res Commun 2005 11 1049 1060 10.1016/j.bbrc.2005.07.005 16039615

[B52] Cicha I Beronov K Ramirez EL Osterode K Goppelt-Struebe M Raaz D Yilmaz A Daniel WG Garlichs CD Shear stress preconditioning modulates endothelial susceptibility to circulating TNF-alpha and monocytic cell recruitment in a simplified model of arterial bifurcations Atherosclerosis 2009 11 93 102 10.1016/j.atherosclerosis.2009.04.034 19481207

[B53] Lin RZ Chang HY Recent advances in three-dimensional multicellular spheroid culture for biomedical research Biotechnol J 2008 11 1172 1184 10.1002/biot.200700228 18566957

[B54] Weidemann A Bernhardt WM Klanke B Daniel C Buchholz B Campean V Amann K Warnecke C Wiesener MS Eckardt KU Willam C HIF activation protects from acute kidney injury J Am Soc Nephrol 2008 11 486 494 10.1681/ASN.2007040419 18256363 PMC2391048

[B55] Giehl K Graness A Goppelt-Struebe M The small GTPase Rac-1 is a regulator of mesangial cell morphology and thrombospondin-1 expression Am J Physiol Renal Physiol 2008 11 F407 F413 18045834 10.1152/ajprenal.00093.2007

[B56] Stahle M Veit C Bachfischer U Schierling K Skripczynski B Hall A Gierschik P Giehl K Mechanisms in LPA-induced tumor cell migration: critical role of phosphorylated ERK J Cell Sci 2003 11 3835 3846 10.1242/jcs.00679 12902401

