# Supplementary figures and images for: HIF-1α activation results in actin cytoskeleton reorganization and modulation of Rac-1 signaling in endothelial cells
Source: Cell Commun Signal. 2013 Oct 21;11:80. doi: 10.1186/1478-811X-11-80 (PMC3895861; doi:10.1186/1478-811X-11-80)

## Supplementary Figure 2

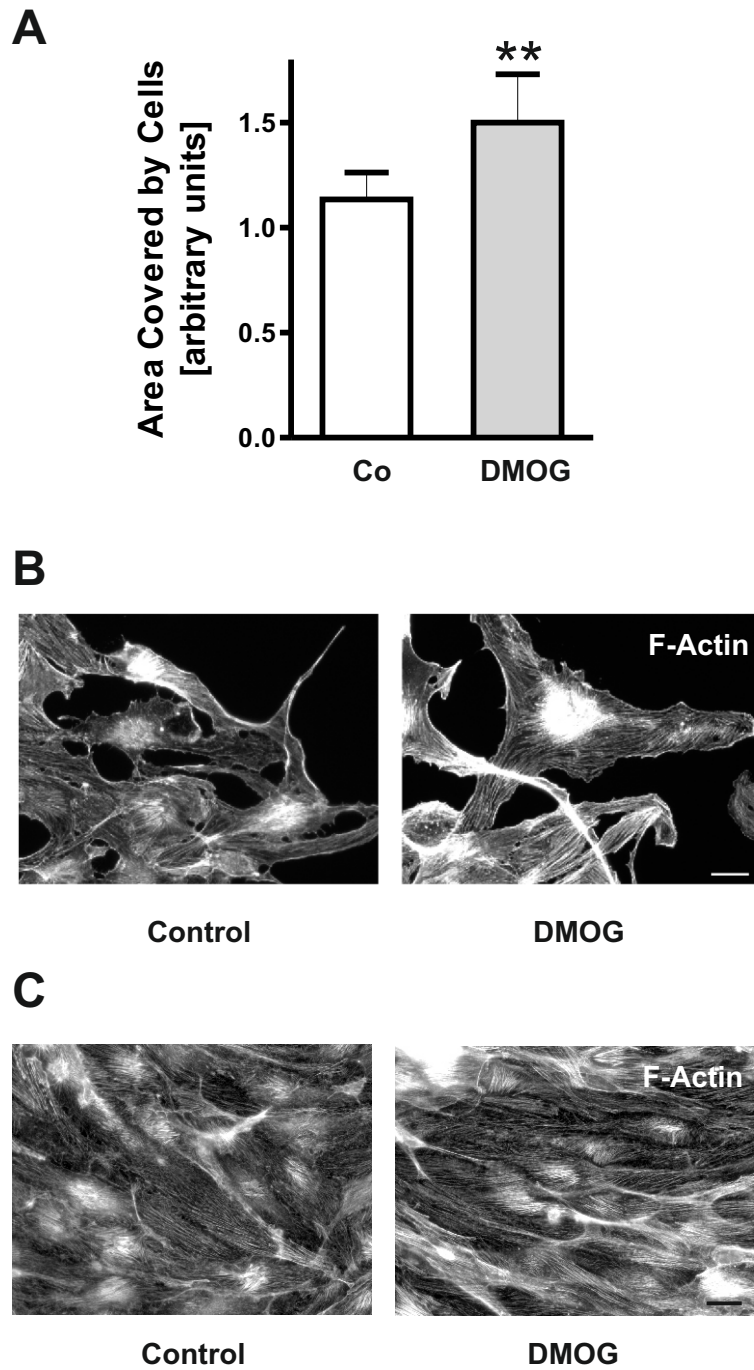

Supplement: Additional file 2 — Figure S2: DMOG-mediated structural alterations in low-density glEND.2. (A) glEND.2 cells were seeded at low density on fibronectin-coated glass plates and incubated for 24 h with or without 1 mM DMOG. Cells were visualized by staining F-actin with PromoFluor phalloidin and staining of nuclei with Hoechst. In each condition, 6 visual fields were randomly chosen, the number of cells was counted and the area covered by the cells determined using ImageJ software. The graph depicts area per cell in arbitrary units, means + SD of 6 fields. ** p < 0.01, Student’s t-test. (B) glEND.2 cells were seeded at low density on fibronectin–coated glass plates and incubated for 24 h with or without 1 mM DMOG. F-actin fibers were visualized by PromoFluor phalloidin staining. DMOG-treated cells showed strong F-actin fibers and appeared more spread. Scale bar: 20 μm. (C) glEND.2 cells were seeded to form a confluent monolayer and then incubated with DMOG for 24 h. F-actin fibers were visualized by PromoFluor phalloidin staining. Scale bar: 20 μm. [file 1478-811X-11-80-S2.pdf]

# Supplementary Figure 3

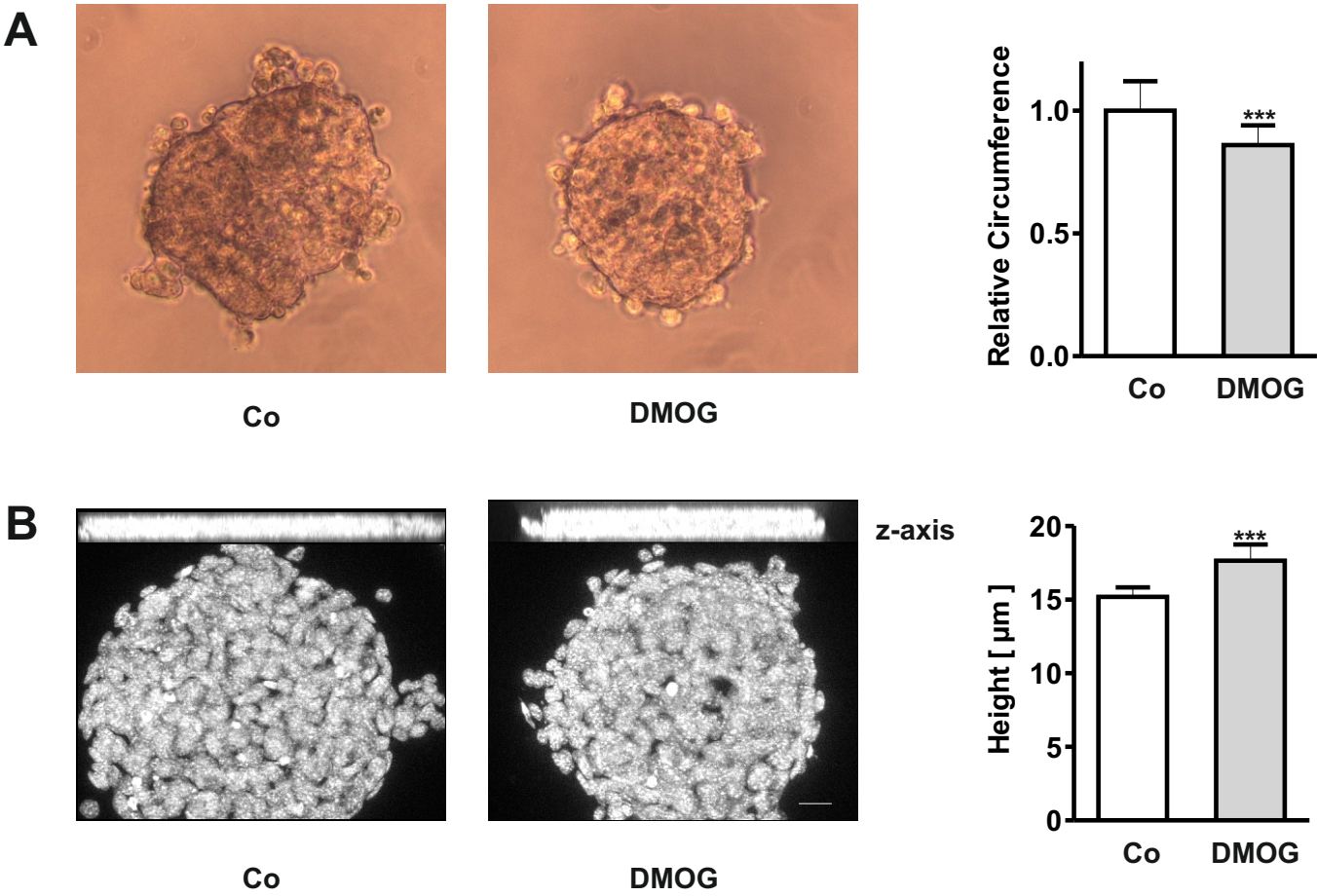

Supplement: Additional file 3 — Figure S3: Generation of spheroids in the presence of DMOG. (A) Spheroids were generated by the hanging drop method in the presence or absence of DMOG. For quantification, spheroids were allowed to adhere on plates for 3 h. Spheroids with DMOG appeared more tightly packed forming round structures compared to the control spheroids which appeared less organized. Accordingly, the area covered by DMOG-treated spheroids was reduced. The graph summarizes data (means + SD) of 30 spheroids of three different experiments. In each experiment the mean value of control spheroids was set to 1, error bars reflect the variability within one experiment. *** p < 0.001, Student’s t-test. (B) After fixation the nuclei were stained with DAPI to assess the height of the spheroids by apotome technique. Layers were merged and the height of the spheroid approximated by the fluorescence of the z-axis. Data of 6 spheroids each of one representative experiment are depicted. *** p < 0.001, Student’s t-test. [file 1478-811X-11-80-S3.pdf]

## Supplementary Figure 4

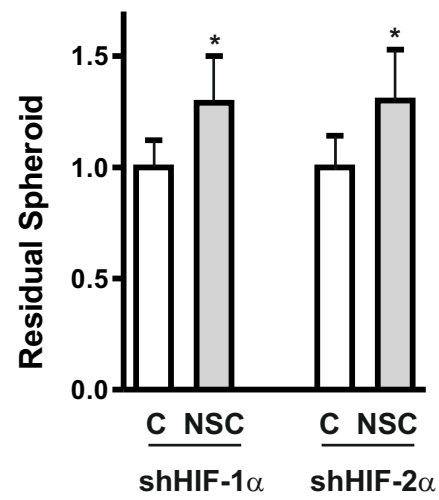

Supplement: Additional file 4 — Figure S4: Inhibition of Rac-1 is effective independently of HIF. Spheroids of shHIF-1 and shHIF-2 clones were treated with 100 μM NSC23677 overnight. The area covered by residual spheroids was determined in 6 spheroids for each condition. Mean value of control cells was set to 1. * p < 0.05, Student’s t-test. The Rac-1 inhibitor increased spheroid size independently of HIF-1 or HIF-2 knockdown. Scale bar: 20 μm. [file 1478-811X-11-80-S4.pdf]
